# The antibacterial efficacy of photon-initiated photoacoustic streaming in root canals with different diameters or tapers

**DOI:** 10.1186/s12903-021-01903-7

**Published:** 2021-10-21

**Authors:** Cheng Wen, Liang Yan, Yuanyuan Kong, Jian Zhao, Yang Li, Qianzhou Jiang

**Affiliations:** Department of Endodontics, Affiliated Stomatology Hospital of Guangzhou Medical University, Guangzhou Key Laboratory of Basic and Applied Research of Oral Regenerative Medical, Guangzhou, 510182 Guangdong China

**Keywords:** Photon-induced photoacoustic streaming, Diameter, Taper, Root canal

## Abstract

**Background:**

In recent years, the concept of minimally invasive endodontics has been proposed, which could be described as preventing or treating diseases by preserving more dental tissue and creating minimal damage. In the process of root canal preparation, it was recommended to use instruments with a smaller taper to preserve more tooth tissue and improve the preservation rate of the affected teeth. Photon-induced photoacoustic streaming (PIPS) was a new type of laser-activated irrigation technology, which was now widely used in endodontic treatment. The purpose of this article was to evaluate the bactericidal effect of PIPS with NaOCl in root canals with different widths or tapers.

**Methods:**

Twenty-three maxillary first molars with three independent root canals were included in this study. The mesiobuccal (MB), distobuccal (DB), and palatal (P) root canals were prepared at sizes of #10/.02, #25/.02, and #25/.06, respectively. After being incubated with a bacterial suspension for 4 weeks, the specimen were irrigated with 2% NaOCl activated by conventional needle irrigation (CNI) (n = 10) or PIPS (n = 10). Three specimen were not treated (control group). Before and after irrigation, the presence of bacteria was assessed with an adenosine 5'-triphosphate (ATP) assay kit and biofilms were assessed using confocal laser scanning microscopy and scanning electron microscopy.

**Results:**

In specimen prepared using PIPS irrigation, the ATP was reduced by more than 98%. When the root canal taper was 0.02, the size #25 root canals had a higher percentage of dead bacteria than the size #10 root canals in all regions (*P* < 0*.*05) in the PIPS group. When the root canal width was #25, the 0.02 taper group had a higher percentage of dead bacteria than the 0.06 taper group in the apical region (*P* < 0*.*05), except coronal and middle regions (*P* > 0*.*05). PIPS irrigation results in a greater percentage of dead bacteria and reduction of ATP in size #10/.02 root canals than CNI in size #25/.06 root canals in three regions (*P* < 0*.*05).

**Conclusion:**

Increasing the width from #10 to #25 improves the bactericidal effect of PIPS in the root canal. Increasing the taper of the root canal from 0.02 to 0.06 at size #25 did not affect the bactericidal effects of PIPS. PIPS resulted in more dead bacteria in specimen with smaller tapers and root canal widths than CNI. PIPS can be used to clean the smear layer in the coronal region and open the dentin tubules.

*Clinical significance*: Activation of irrigants with PIPS brought about significant bacterial reduction smaller tapers and width root canals compared to CNI, which was beneficial to prevent excessive loss of tooth tissue and conserve the structural integrity of teeth.

## Background

Complete elimination of soft tissues, infected debris, and microorganisms remains a challenge during root canal treatment [1]. Due to the intricate anatomy of root canals, an area of the canal remains untouched during canal preparation. This may continue to exist in the root canal system, leading to pulp disease after treatment [2]. Chemomechanical preparation is a crucial step for a successful root canal treatment. Mechanical preparation and irrigating solutions play key roles in providing debridement and removing vital and necrotic tissue, debris, and microorganisms from the root canal system [3]. Studies have shown that a larger root canal preparation width and taper can improve the effectiveness of root canal irrigation; however, optimization of the mechanical efficacy of irrigation provided by enlarging the canals may result in weakening of the root structure [4] and increase the risk of root fracture [5].

Minimally invasive endodontics (MIE) has been recently introduced and can be used to prevent or treat diseases by preserving tissue and creating minimal damage [6]. The use of instruments with smaller tapers during root canal preparation has been proposed to preserve more dentine and decrease stress, mainly on the coronal third of the teeth undergoing a root canal treatment [7, 8]. However, it is also known that irrigating minimally-enlarged canals may also pose additional disadvantages such as limited irrigant penetration, needle wedging, the vapor lock effect, and challenges associated with sonic/ultrasonic/apical negative pressure irrigation [9]. The main concern when using instruments with smaller tapers are their ability to clean and shape root canals. Therefore, it is important to find a more effective instrument for cleaning root canals during root canal disinfection.

Photon-induced photoacoustic streaming (PIPS) is a new laser agitation technique. In this technique, the laser is based on the creation of cavitation phenomena and acoustic streaming in intracanal fluids related to the photomechanical effects of lasers at low settings [10]. PIPS differs from other techniques in that the working head tip is placed in the coronal portion of the root canal, avoiding heat damage to the wall of the canal and the periapical tissue. Our previous study reported that PIPS combined with 2% NaOCl results in a strong bactericidal effect on a straight root canal model [11]. In the present study, we used an adenosine 5'-triphosphate (ATP) assay kit, confocal laser scanning microscopy (CLSM), and scanning electron microscopy (SEM) to evaluate the bactericidal effect of PIPS with NaOCl in different root canals with different widths and tapers. The null hypothesis of the test was that increasing the taper and diameter of the root canal had no positive effect on improving the irrigating solution's ability to remove bacteria.

## Methods

### Specimen selection

The study was approved by the ethics board of the School and Hospital of Stomatology, Guangzhou Medical University. A previous study [12] was used to identify an effect size of 0.50 required to calculate the total sample size for this study. α-type error = 0.05 and power (1 − β) = 0.80 were also inputted (F test family, ANOVA, G*Power for Windows). A minimum of 10 teeth per group should be used. Three groups were positive control groups. Therefore, a total of 23 extracted maxillary first molars with three independent dental roots and no visible root caries, cracks, or root resorption under dental microscope (Zumax, Suzhou, China) were selected. The teeth were extracted from a Chinese population, and ultrasonically cleaned and stored in a sterile saline solution for no more than 48 h.

### Root canal preparation

Prior to treatment, part of the crown was removed using high-speed diamond burs so that a working length of 19 mm was maintained in each specimen. The apical foramen was sealed with Beautifil Flow Plus F00 (SHOFC, Japan). The pulp chamber was opened and examined under a microscope to determine the root canal orifice. The mesiobuccal (MB), distobuccal (DB), and palatal (P) root canals were prepared to #10/.02, #25/.02, and #25/.06 respectively.

In the #10/.02 group, the MB root canal was prepared using a #10/.02 K-file (Dentsply, Switzerland). It was sized to the working length, and a reciprocal action was used until it fit loosely in the canal. In the #25/.02 group, the DB root canal was prepared using a #25/.02 K-file. A #10/.02 K-file was used, followed by a #15/.02 K-file, #20/.02 K-file, and #25/.02 K-file until it fit loosely in the canal. In the #25/.06 group, the P root canal was prepared using MTWO rotary files (VDW, Munich, Germany) and an electric motor (VDW). The following files were used: #10/.04, #15/.05, #20/.06, and #25/.06. The instrument was used to the full length of the canals (a single-length technique) with a gentle in-and-out motion until the working length was reached.

Each file was used to enlarge one canal only. All root canal preparations were completed by one operator.

### Bacterial inoculation of the root canals

*Enterococcus faecalis* (ATCC 29212) was grown in brain heart infusion (BHI) broth (Hopebio, Qingdao, China). Single colonies were inoculated with 5 mL of BHI in an aerobic chamber for 24 h at 37 °C. A 5 × 10^8^ CFU/mL suspension, equivalent to .5 McFarland, was prepared. Then, 200 μL of the bacterial suspension was added to the root canal systems. The specimens were incubated for 4 weeks at 37 °C, and the bacterial suspension was refreshed every 24 h. After incubation, the root canals were washed with 1 mL distilled water. Three infected teeth were randomly selected for the control group and were untreated. The remaining 20 teeth were divided into two groups. Sterile paper points were inserted into the root canal system and left in the canal for 1 min to collect planktonic bacteria. Samples from each canal were collected using separate Eppendorf tubes (S0 sample). Before irrigation of the root canals, three infected samples were randomly selected to measure the ATP value and the percentage of dead bacteria using the ATP assay kit and CLSM, respectively.

### Root canal irrigation

Conventional needle irrigation (CNI) was performed for 10 specimen. The 30-gauge irrigation side-vented needle tip was placed 3 mm below the root canal orifice in the #10/.02 group and at the working length in the #25/.02 and #25/.06 groups. No tips were in contact with the root canal wall. The root canals were irrigated for 60 s with 3 mL of 2% NaOCl.

PIPS was performed for 10 specimens. The irrigation tip remained stationary in the pulp chamber and was activated for the cycles described above while ensuring that the canal and pulp chamber remained passively filled with the irrigating solution throughout the irrigation. The irrigant was activated by a 2,940 nm Er YAG laser (AT Fidelis; Fotona, Ljubljana, Slovenia) equipped with a handpiece (R14-PIPS, Fotona) with a 400-μm-diameter quartz tip (XPulse 400/14, Fotona). The tip was applied at 0.3 W, 15 Hz, and 20 mJ per pulse, as recommended by the manufacturer, without water/air spray [13]. The fiber tip was placed in the pulp chamber. Irrigation in the root canals was activated for 30 s with 3 mL of 2% NaOCl.

After CNI or PIPS, all samples were irrigated with 1 mL of distilled water to remove residual irrigating solution from the root canal. Then, sterile paper points and Eppendorf tubes were used to collect the S1 samples. The above processes were completed by an experienced single operator of the dental pulp department. Each file was used to prepare one root canal system.

### ATP assay kit analysis

The ATP in the root canal system was measured as previously reported [14] using an ATP assay kit (Beyotime, China, S0026) according to the manufacturer's instructions. Each sample of bacteria was collected via the sequential placement of sterile paper points in the root canal before irrigation (S0) and after irrigation (S1). The paper points were placed in the root canal for 1 min to obtain a sample of bacteria and then transferred to a 1.5-mL Eppendorf tube containing 200 μL of lysis buffer with 0.025 g of glass beads (D3350-01, Omega Biotek Inc., America), and centrifuged at 12,000 g/min for 5 min at 4 °C to collect the supernatant. An ATP detection solution was then prepared. The ATP detection solution (20 μL) and 80 μL of diluted solution were mixed in a detection tube and incubated at room temperature for 5 min. Then, 20 μL of the sample was added to the ATP detection solution and quantified using an ATP fluorescence detector (Lux-T020, China).

### CLSM analysis

CLSM analysis was performed to distinguish viable and nonviable bacteria on the root canal walls and in the dentin tubules. Longitudinal grooves were carved onto the root surfaces with high-speed diamond burs without entering the inner parts of the root canals. A chisel was then used to split the tooth open into two pieces that were placed in 1 mL tubes. Then, one half of the root surface was exposed to the reagents of a LIVE/DEAD BacLight Bacterial Viability Kit (L7012, Life, USA) for 15 min, per the manufacturer’s instructions. The apical, middle, and coronal thirds were established by marking the roots at 0–3, 3–6, and 6–9 mm from the apical foramen. A CLSM (Carl Zeiss, Germany) was used to detect the presence of green biofilms (living) or red biofilms (dead) on the root canal walls and in dentin tubules with a 20 × objective lens. The biofilm images of each scanning area were acquired at a step size of 10 μm, and a total area of 100 μm was scanned, resulting in 10 layers. Zen Black software (Carl Zeiss, Germany) was used to perform the 3D reconstruction of each layer.

### SEM analysis

SEM analysis was performed to determine the presence of bacterial infection in canal cross-sections and bacterial penetration inside the dentin tubules. The remaining half of the root surface that did not undergo CLSM analysis was fixed in 2.5% glutaraldehyde for 24 h, dehydrated in an ascending acetonitrile series (50%, 70%, 80%, 90%, and 100% twice for 20 min each), dried at room temperature, sputter-coated with platinum (Ion Sputter E-1045; Hitachi), and observed using SEM (S-4800; Hitachi).

### Statistical analysis

All statistical analyses were performed using SPSS version 17.0 statistical software (IBM SPSS Inc, Chicago, IL, USA). The normality of the data distribution was assessed. Normally-distributed data are presented as mean and standard deviations (SD) and compared using a one-way analysis of variance (ANOVA). Statistical significance was set at *P* < 0.05.

## Results

**Fig. 1:**
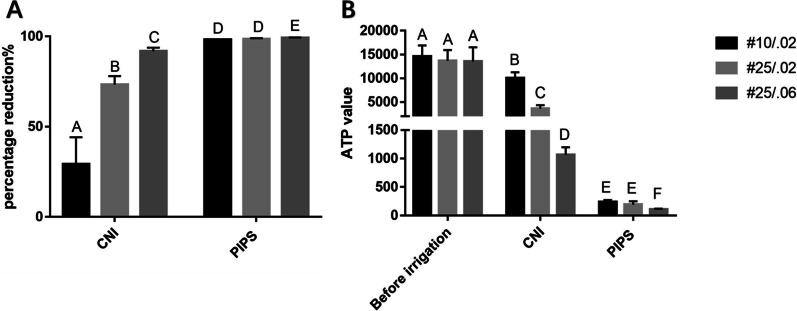
The ATP value in the root canal systems after irrigation. **A** The reduction of ATP after conventional needle irrigation (CNI) or photon-induced photoacoustic streaming (PIPS) is shown. The values are presented as mean and standard deviation. **B** The ATP values before and after irrigation are shown as mean and standard deviation. Statistical significance is represented by uppercase letters

**Fig. 2:**
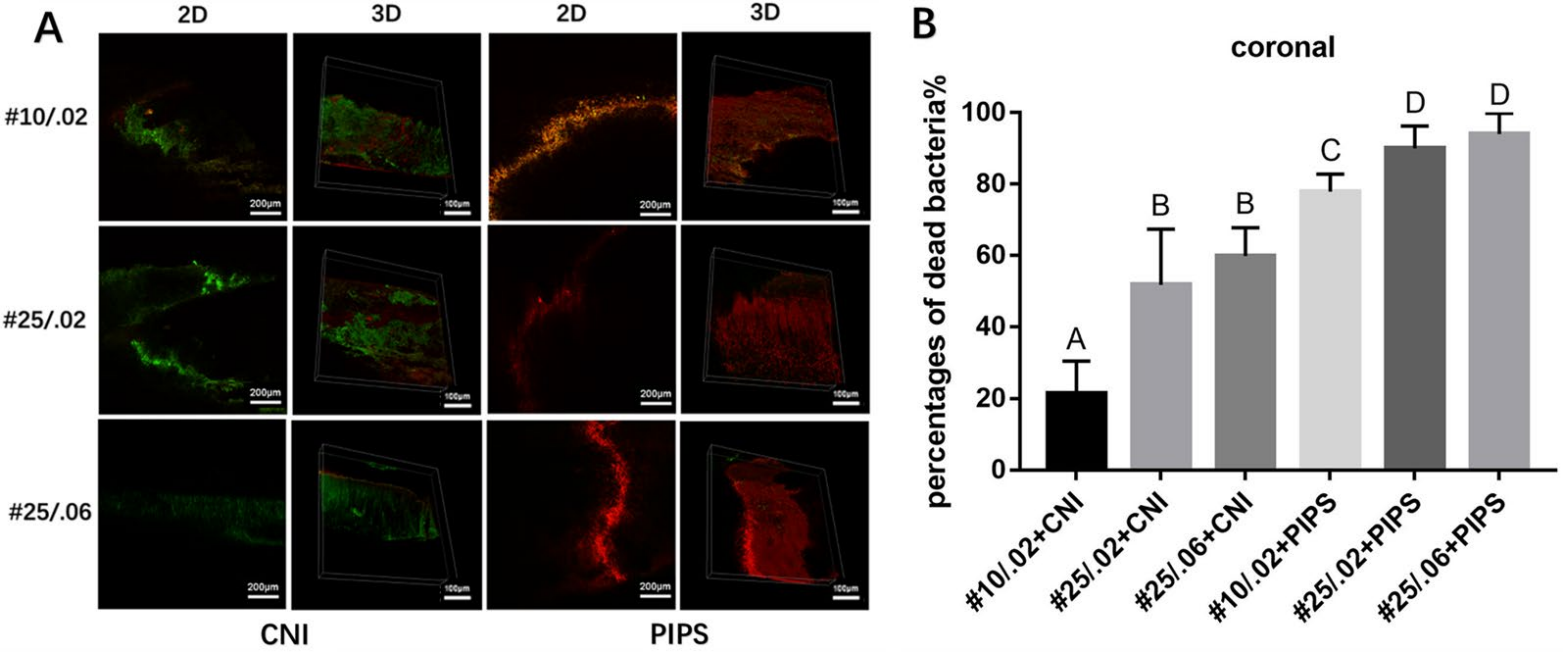
Representative CLSM images of live or dead *E. faecalis* biofilms on the canal walls in the coronal region of root canals after irrigation. **A** The left and middle-left images of each row represent 2D and 3D structures of biofilms in the coronal region irrigated by conventional needle irrigation (CNI). The right and middle-right images represent 2D and 3D structures of biofilms in the coronal region irrigated by photon-induced photoacoustic streaming (PIPS). **B** The percentages of dead bacteria in *E. faecalis* biofilms are shown. The values are shown as mean and standard deviation. Statistical significance is represented by uppercase letters

**Fig. 3:**
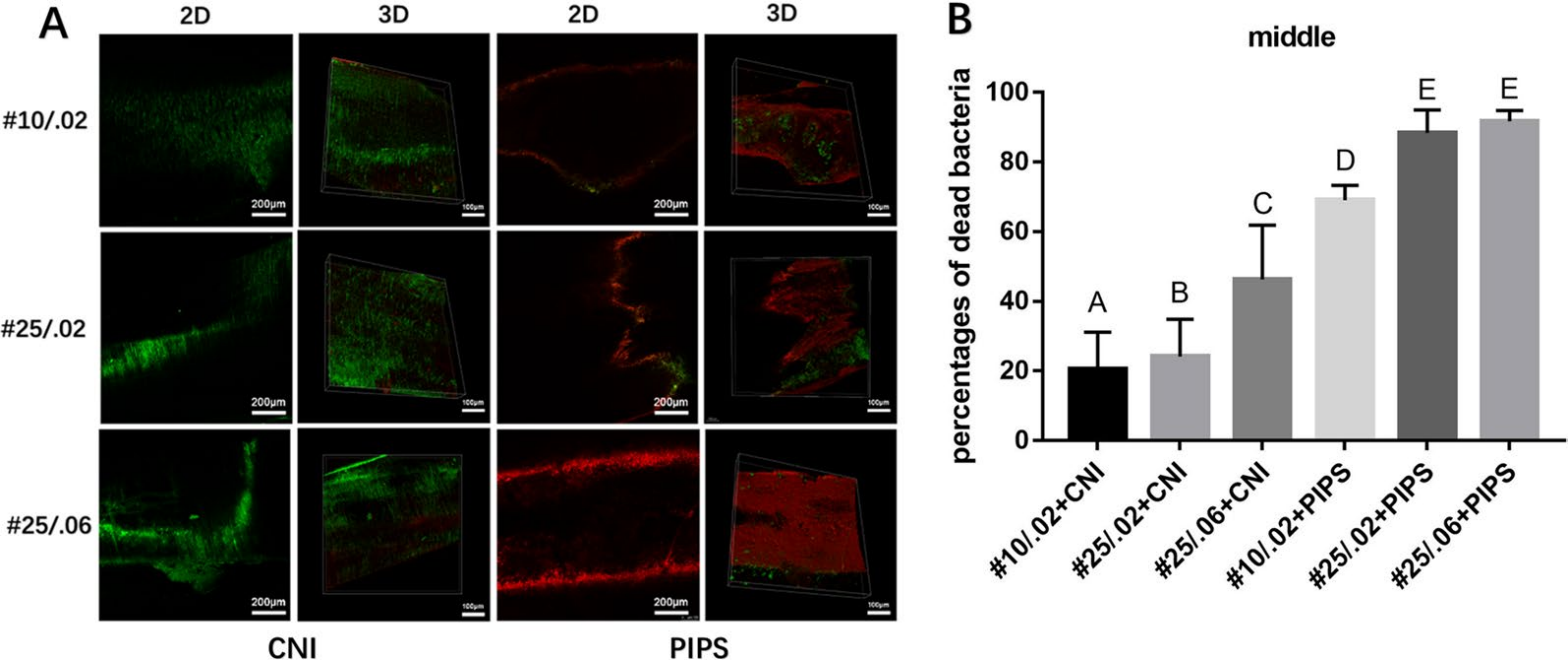
Representative CLSM images of live or dead *E. faecalis* biofilms on canal walls in the middle region of root canals after irrigation. **A** The left and middle-left images of each row represent 2D and 3D structures of biofilms in the middle region irrigated by conventional needle irrigation (CNI). The right and middle-right images represent 2D and 3D structures of biofilms on in the middle region irrigated by photon-induced photoacoustic streaming (PIPS). **B** The percentages of dead bacteria in *E. faecalis* biofilms are shown. The values are shown as mean and standard deviation. Statistical significance is represented by uppercase letters

**Fig. 4:**
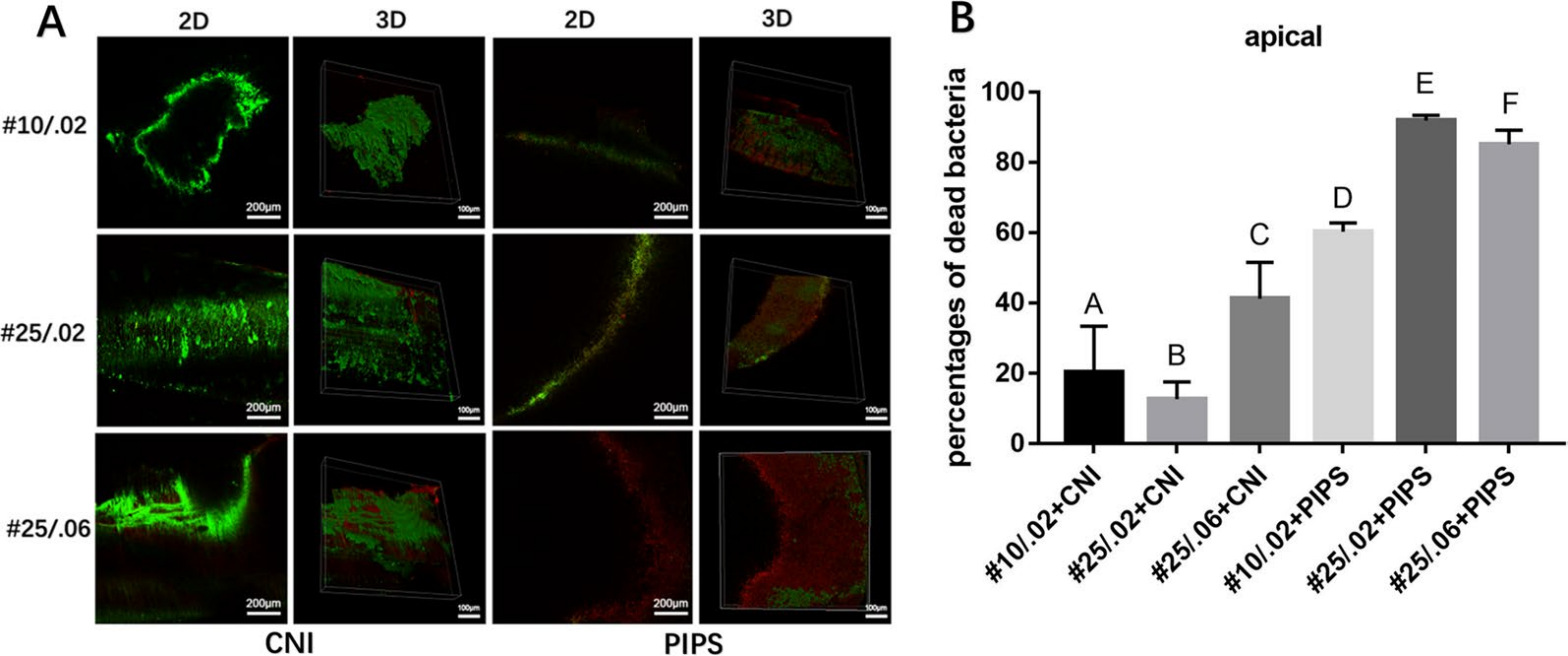
Representative CLSM images of live or dead *E. faecalis* biofilms on canal walls in the apical region of root canals after irrigation. **A** The left and middle-left images of each row represent 2D and 3D structures of biofilms in the apical region irrigated by conventional needle irrigation (CNI). The right and middle-right images represent 2D and 3D structures of biofilms on in the apical region irrigated by photon-induced photoacoustic streaming (PIPS). **B** The percentages of dead bacteria in *E. faecalis* biofilms are shown. The values are shown as mean and standard deviation. Statistical significance is represented by uppercase letters

**Fig. 5:**
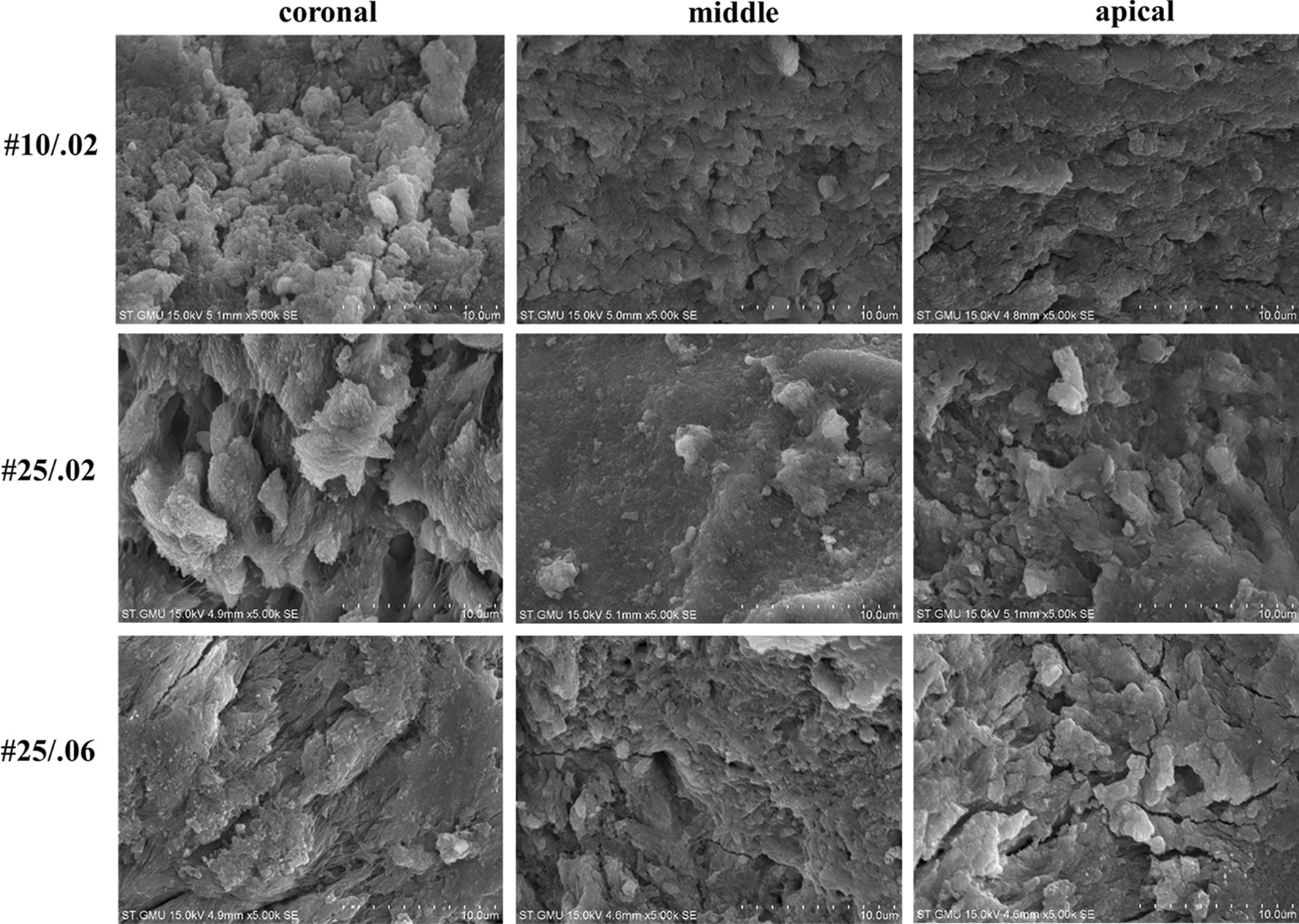
Scanning electron microscopy (SEM) images in the coronal, middle, and apical thirds of root canals irrigated using conventional needle irrigation (CNI) are shown

**Fig. 6:**
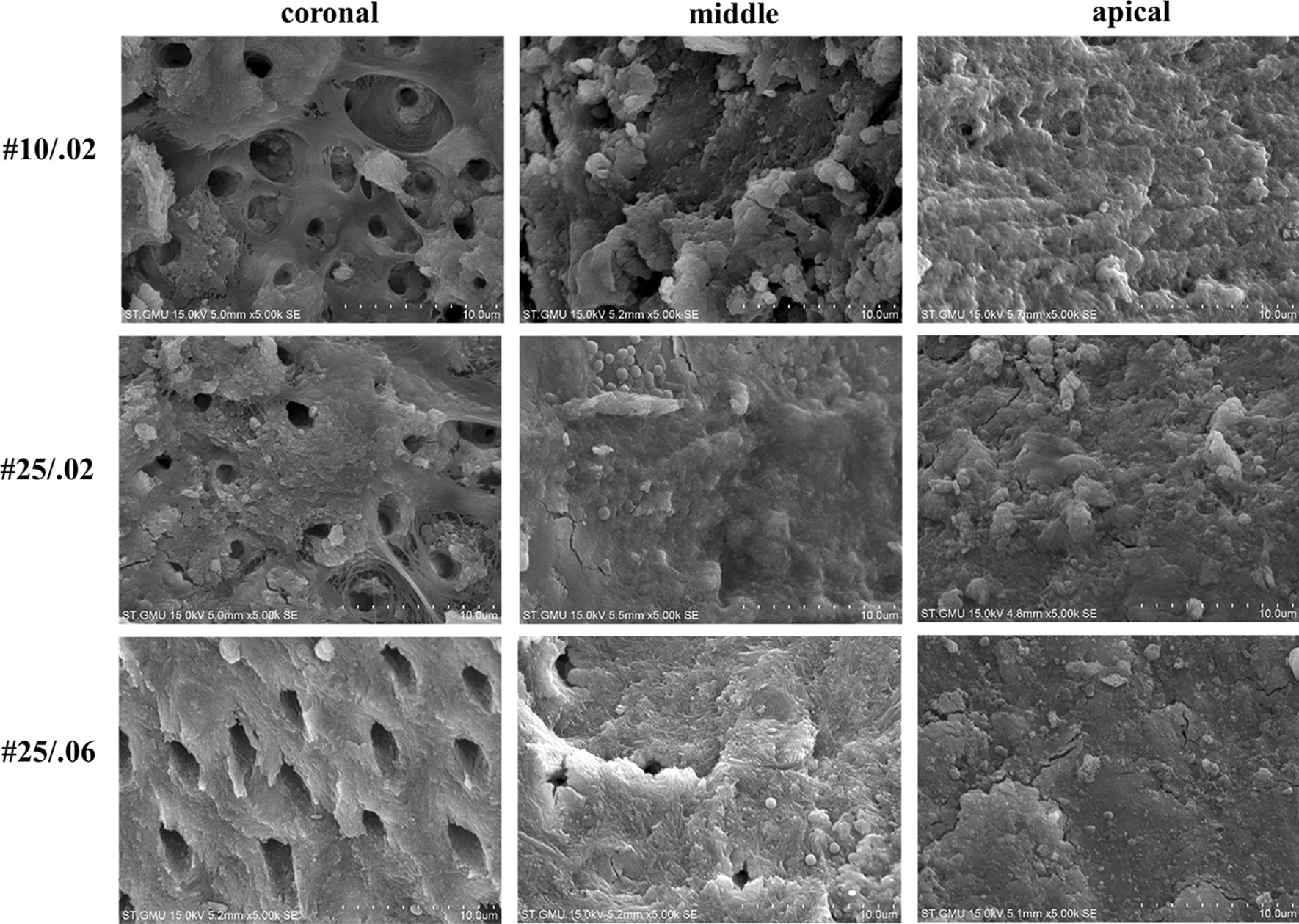
Scanning electron microscopy (SEM) images in the coronal, middle, and apical thirds of root canals irrigated using photon-induced photoacoustic streaming (PIPS) are shown

Prior to root canal irrigation, the ATP value and percentage of dead bacteria were not statistically different between the groups (*P* > 0*.*05). The median ATP value for whole canals is shown in Fig. 1, and the median percentages of dead bacteria in the coronal, middle, and the apical regions are shown in Figs. 2, 3, and 4, respectively. Representative SEM images of the coronal, middle, and apical thirds of all groups after irrigation with CNI or PIPS are presented in Figs. 5 and 6, respectively.

### ATP analysis

In both the CNI and PIPS groups, a reduction in the ATP value after irrigation was observed, though PIPS resulted in a greater reduction of ATP. After PIPS, ATP was reduced by 98.36% ± 0.21% in size #10/.02 root canals, by 98.56% ± 0.43% in size #25/.02 root canals, and by 99.18% ± 0.25% in size #25/.06 root canals. The ATP reduction in size #10/.02 root canals was significantly greater after PIPS than after CNI (91.79% ± 1.85%) (*P* < 0*.*05)*.*

### CLSM analysis

In the CNI group, the percentage of dead intracanal bacteria was highest in root canals sized #10/.02, followed by #25/.02, and #25/.06 (*P* < 0*.*05). In the PIPS group, root canals sized #25/.02 had a significantly higher percentage of dead bacteria than root canals sized #10/.02 in all three regions (*P* < 0*.*05)*.* Root canals sized #25/.02 had a significantly higher percentage of dead bacteria than root canals sized #25/.06 (*P* < 0*.*05) in the apical region, but no significant differences were observed between these sizes in the coronal or middle regions.

The percentage of dead bacteria was significantly higher in the #10/.02 root canals that underwent PIPS compared to the #25/.06 root canals that underwent CNI in all three regions (*P* < 0*.*05).

### Scanning electron microscopy

Open dentin tubules were observed in the root canal system activated by PIPS in the coronal region. A smear layer and debris were observed on the root dentin in other regions.

## Discussion

MIE has recently attracted attention from clinicians. As a vital part of MIE, preserving dental hard tissue is considered a practical method to increase the long-term success of endodontically-treated teeth [15]. Therefore, disinfection is particularly important during the mechanochemical preparation of root canals.

Bacteria within biofilms are tenfold to 1,000-fold more resistant to antimicrobial agents and antibiotics than planktonic (free-living) bacteria and are also able to effectively evade the immune system [16]. This ex vivo study used a monospecies biofilm model of *E. faecalis*, which is a gram-positive microorganism commonly detected in asymptomatic, persistent endodontic infections. This microorganism exists in the biofilm of the root canal as planktonic bacteria [17]. Planktonic bacteria can attach to the wall of the root canal to form a biofilm, and the biofilm can also detach to form planktonic bacteria [18]. Biofilms are defined as highly organized structures consisting of bacteria enclosed in a self-produced extracellular matrix attached to a surface [19].

Our results suggest that PIPS has a greater bactericidal effect than CNI for planktonic bacteria and bacteria within biofilms in root canal systems after irrigation. This is consistent with the results of previous studies [20, 21]. Do and Gaudin [22] conducted a review of 59 studies related to PIPS and determined that 18 studies addressed canal disinfection. Two-thirds of these studies (11/18) concluded that PIPS is effective in killing bacteria, significantly more than CNI. The greater bactericidal effects may be attributed to the laser energy used in PIPS, which exhibits the highest absorption by water and hydroxyapatite of any laser, resulting in a three-dimensional stirring fluid throughout the root canal [23]. Laser activation was achieved by cavitation, where vapor bubbles that expanded and collapsed were formed at the fiber tip [10]. These large elliptical bubbles implode after 100 to 200 µs, inducing the secondary cavitation effect to remove planktonic bacteria and biofilms in root canal systems [24].

The volume of the root canal after preparation in #25/.06 was significantly larger than that in #10/.02 and #25/.02. The larger the volume of the culture medium was, the greater the bacterial growth. *E. faecalis* could form a stable biofilm model in root canals after 21 days of culture [25]. Three independent roots could ensure that there were no communicating branches in each root canal, which could affect the bacterial culture and flushing effect, but the same culture condition could ensure the consistency of biofilms formed in each root canal after 21 days of culture. Prior to root canal irrigation, the ATP value and percentage of dead bacteria were not statistically different between the groups (*P* > 0*.*05).

After CNI, the percentages of dead intracanal bacteria were highest in #10/.02 root canals, followed by #25/.02, and #25/.06. This may be because when using CNI + 1% NaOCl, the larger the root canal volume, the more irrigation solution in the root canal, which was more conducive to irrigation solution replacement and better sterilization effect. Our results are consistent with those of previous studies [26, 27]. Deeper irrigate penetration is desirable, as some ex vivo experiments have demonstrated that CNI only allows for NaOCl infiltration up to 250 µm into the root dentine [28]. Therefore, a large number of green living bacteria were observed on CLSM.

In the PIPS group, when the taper was 0.02, size #25 root canals had a higher percentage of dead bacteria than size #10 root canals in all three regions. The ATP in #10/.02 and #25/.02 root canals was reduced by more than 98%*.* These results suggest that enlarging the width of the root canal from #10 to #25 improves the bactericidal effect of PIPS in the root canal when the same taper is used.

Previous studies have reported that large taper instruments have a better debridement effect on the coronal and middle regions of root canals [29, 30]. In this study, when the root canal width was #25, there were no significant differences between the root canals with a taper of 0.02 and those with a taper of 0.06 in the coronal and middle regions when PIPS was used. In the apical region, #25/.02 root canals had a significantly higher percentage of dead bacteria than #25/.06 root canals after PIPS. These results indicate that enlarging the taper of root canals had no positive effect on the removal of biofilms with PIPS with 2% NaOCl. Peeters and Mooduto [31] reported that using a PIPS laser tip in the coronal region of root canals may drive the irrigating solution to the end of the canal with no adverse effects on the apical tissues. Therefore, PIPS may achieve a better cleaning effect without enlarging the apical taper or damaging periapical tissue, which is clinically important.

In all three regions, #10/.02 root canals that underwent PIPS had a significantly higher percentage of dead bacteria than #25/.06 root canals that underwent CNI. These results indicate that PIPS has a better bactericidal effect than CNI in root canals with a smaller taper and a smaller width. This may be because PIPS improved the replacement speed of the irrigation solution, which could effectively sterilize the root canal even if the volume was small in root canal system. Cheng et al. [32] reported that the disinfection efficacy of PIPS with NaOCl in size #15 root canals was similar to that of CNI with NaOCl in size #40 root canals (*P* > 0*.*05). In other words, the same disinfection efficacy was achieved with PIPS using a much smaller width compared to CNI. The preparation of root canals with a smaller taper and width may be useful to prevent excess loss of dental tissues and conserve the structural integrity of endodontically-treated teeth, and may be a promising procedure for MIE [33–36]. Therefore, the null hypothesis was rejected.

The highest percentage of dead bacteria in the PIPS group was in the coronal region, followed by the middle region, and the apical region. SEM imaging revealed open dentin tubules in the coronal regions of root canal systems that underwent PIPS. A smear layer and debris were observed on the root dentin in other regions. This may be due to the fact that we did not add ethylenediaminetetraacetic acid (EDTA) to remove the smear layer during the root canal preparation. In the PIPS group, the coronal dentin tubules were observed to be open, indicating that PIPS was able to remove the bacterial biofilm and smear layer. Jiang et al. [37] reported that the PIPS tip is placed only in the coronal region of the specimen, and its photoacoustic shock wave may weaken over the distance to the apical region; therefore, the effectiveness of PIPS in removing bacterial biofilms from the apical regions is less than that of the coronal region. Our results are consistent with these previous studies.

The limitation of this study is that the use of *E. faecalis* alone for evaluation will oversimplify the biofilm model, and there are large differences between single-species and multi-species biofilms. Despite discrepancies in the methodology used in previous studies, most of the results indicate that PIPS is an effective bactericidal method during root canal preparation. The results of our study clearly indicate that PIPS significantly improved antimicrobial action against *E. faecalis* planktonic bacteria and biofilms in vitro. PIPS, which works by inserting the laser tip into the coronal third of the canal, was more effective than CNI for smaller root canal systems in this study. These findings indicate that PIPS may be a highly promising laser application in endodontics.

## Conclusion

Increasing the width of the root canal improves the bactericidal effect of PIPS when the same taper is used. Increasing the taper had no positive effect on the antibacterial effect of PIPS in #25 root canals. PIPS had a greater bactericidal effect in root canals with a smaller taper and width. PIPS effectively cleaned the smear layer and opened the dentin tubules in the coronal region of the root canals.

## Data Availability

The raw data are available from the authors to any researcher who wishes to collaborate with us.

## Abbreviations

PIPS: Photon-initiated photoacoustic streaming
CNI: Conventional needle irrigation
ATP: Adenosine 5'-triphosphate
BHI: Brain heart infusion
